# HOW AN INDIVIDUAL’S SENSE OF CONTROL INFLUENCES THEIR PERCEPTION OF SUCCESSFUL AGING

**DOI:** 10.1093/geroni/igz038.1235

**Published:** 2019-11-08

**Authors:** Alexandra P Harris, Alexandria Nuccio, Ashely M Stripling

**Affiliations:** 1 Nova Southeastern University, Fort Lauderdale, Florida, United States; 2 Nova Southeastern University, Fort Lauderdale, United States

## Abstract

Factors like physiology, mental health, personal resources, and social support have been identified to contribute to perceived successful aging (Cosco, Prina, Perales, Stephan & Brayne, 2015); however, sense of control’s role in these relationships remains underexplored. Studying the impact of sense of control is crucial, given that many factors associated with well-being are correlated with later life success. The current study investigates associations among sense of control and constructs known to define successful aging. The data was derived from the Survey of Midlife in the US database (MIDUS3). Participants were primarily Caucasian (88.7%) and female (54.9%) with a mean age of 63.64 years (SD=11.35). A series of hierarchical multiple regressions revealed that sense of control impacts physical health (F = 87.734, p<0.001), depression (F =43.944, p<0.001), anxiety (F =24.680, p<0.001), social actualization (F = 66.450, p<0.001), and instrumental activities of daily living (IADL) (F = 135.963, p<0.001) over demographic correlates (i.e., age, sex, and race). The present findings suggest that higher levels of control results in increased comfort in social atmospheres, absence of mood symptoms, good health, and limited issues with IADL. Implications of the current findings include a deeper understanding of how psychological factors, such as sense of control, can impact physical and mental health in order to improve care and promote wellbeing in late life.

